# Assessment of serum Vit A, D and zinc nutritional status and related dietary and exercise behaviors of children and adolescents in rural and urban area

**DOI:** 10.3389/fnut.2022.1088155

**Published:** 2023-01-12

**Authors:** Yan Zou, Li-chun Huang, Dong Zhao, Meng-jie He, Danting Su, Rong-hua Zhang

**Affiliations:** Zhejiang Provincial Center for Disease Control and Prevention, Hangzhou, China

**Keywords:** vitamin A, vitamin D, children, adolescents, rural area

## Abstract

Vitamin A and vitamin D deficiency in children and adolescents has a negative impact on their growth and development. This study aimed to learn the nutritional status of vitamin A and D among rural children and adolescents and to explore related dietary and exercise behaviors. A total of 10 counties (cities, districts) from 90 counties (cities, districts) in Zhejiang Province were selected by the method of random cluster sampling. Children and adolescents were investigated and their food and nutrient intake were calculated. The concentration of serum vitamin A in urban area was 0.38 ng/ml, which was higher than that in rural area (*p* < 0.05); while the concentration of serum vitamin D in urban area was 21.25 mg/L, which was lower than that in rural area (*p* < 0.05). The concentration of serum zinc was 101 μg/dl in urban area and 107 μg/dl in rural area (*p* < 0.05). The intake of dietary fiber, calcium, vitamin A, vitamin B1, vitamin B2, and vitamin C was lower than the recommended value. In rural area, the intakes of cereals, tubers and beans, livestock, poultry and meat of children and adolescents were higher than the recommended values; while the intake of vegetables, fruits, eggs, milk, fish and shrimp, soybean and nuts was lower than the recommended value. The intake of edible oil and salt is higher than the recommended value. The time of medium and high intensity exercise time in rural area is more than that in urban area in the age group of 12–17 years, while the sitting time is less than that in urban area. Children and adolescents living in rural area should be guided to eat reasonably, and to choose foods with high nutrient density and with low oil, salt and sugar.

## Introduction

Today, nearly one in three persons globally suffers from at least one form of malnutrition: wasting, stunting, vitamin and mineral deficiency, overweight or obesity and diet-related non-communicable diseases. Whilst we have seen a decrease in stunting we have seen an increase in all other forms of malnutrition (1). Micronutrients are vitamins and minerals needed by the body in very small amounts. Micronutrient deficiencies can’t only cause visible and dangerous health conditions, but also lead to reduced educational outcomes, reduced work productivity and increased risk from other diseases and health conditions. Deficiency of vitamin A causes blindness in severe cases, and deficiency of vitamin D has well-defined classical functions related to metabolism and bone health (2, 3). Since vitamin A cannot be synthesized by the body, people of all ages can be affected by the deficiency of vitamin A (4). Chronic low intake of vitamin A from diets is the main underlying cause of vitamin A deficiency (VAD), especially in nutritionally demanding periods of life (5). Whereas the adverse effects of restricted intakes of protein, fat, and carbohydrate on physical performance are well-known, there is limited information about the impact of low intakes of micronutrient on the exercise capacity and performance of humans. Physically active people generally consume amounts of vitamins and minerals consistent with the recommendations for the general public. China exhibits a high prevalence of vitamin D deficiency among children and adolescents, caused by factors such as poor economic status and dietary habits, especially in rural areas (6). Food sources of vitamin D are limited (i.e., seafood, eggs, milk and dairy products, meats, and mushrooms), and the ultraviolet exposure is generally low (7). A meta-analysis showed that children and adolescents with obesity have higher risk of vitamin D deficiency (8). And vitamin D is positively associated with physical activity (9).

Evidence for the importance of zinc for all immune cells and for mounting an efficient and balanced immune response to various environmental stressors has been accumulating in recent years (10). Despite a heterogeneous distribution in the body, zinc has no clear storage compartment, which is why an organism is dependent on a daily intake. Good sources of Zinc include beef, pork, cheese, milk, and eggs. Vegetable zinc sources are nuts. According to estimates, 17% of the world population is at risk of insufficient zinc intake (11).

However, the current status of serum Vit A, D and zinc nutritional status and related dietary and exercise behaviors of children and adolescents has rarely been explored. The present study aimed to assess serum Vit A, D and zinc nutritional status and related dietary and exercise behaviors of children and adolescents in rural and urban area. The current analysis will help to inform potential intervention targets and strategies of improving the situation of micronutrient deficiencies and reducing malnutrition for children and adolescents living in rural area.

## Materials and methods

### Study design and participants

This study was based on data obtained from the China National Nutrition and Health Survey 2016–2017 (CHNNS2016-2017). Our study chose children and adolescents from 10 investigation sites in Zhejiang Province including urban and rural areas to form provincial representative sample of Zhejiang Province to carry out food and nutrient analysis and micronutrient deficiency influencing factor exploring. A total of 1,421 children and adolescents aged 6–17 years were actually investigated, including 714 males and 707 females.

Ethics approval was obtained from the Ethical Committee of Zhejiang Provincial Center for Disease Control and Prevention. All student guardian provided written informed consent after the research protocols were carefully explained to them. Thus, informed consents from the parents/guardians of all participants were received.

### Social-demographic characteristics and measurement

General information including gender, date of birth, and living area were collected by questionnaire. Exercise behaviors were also collected with questionnaire concentrated on medium and high intensity exercise time and sitting time. The medium and high intensity exercise refers to the physical activity with shortness of breath and fast heart rate, but language communication can be carried out and subjective feeling is slightly laborious. Sitting time refers to the total time without any physical activity or with very little physical activity during work, housework, transportation, or leisure time.

Anthropometrical measurements were conducted by well-trained health workers of local community health center who followed a reference protocol recommended by the WHO (12). Height was measured without shoes to the nearest 0.2 cm using a portable stadiometer (TZG, Wuxi weighing instrument factory Co., Ltd., Wuxi, China), and weight was measured without shoes and in light clothing to the nearest 0.1 kg on a calibrated beam scale (G&G tc-200k, Shanghai taizhiheng electronic weighing instrument Co., Ltd., Shanghai, China). BMI was calculated by weight (kg)/height(m)^2^. According to “Screening for overweight and obesity among school-age children and adolescents” (13), we judged overweight and obesity of children aged 6–17 years by BMI boundary values which are stratified by age and gender.

### Dietary assessment

During home and school visits spanning 3 days, dietary data were collected through interviews with each child of the sampling schools, including breakfast, lunch, dinner and the intake of extra meals or snacks. All the foods and condiments and edible oil that intake during the three consecutive days of 24-h were recorded. Combined with the China Food Composition Table published in 2002 (14), energy and nutrient intake were calculated. According to nutrition guidelines of school meals (nutrition guidelines of school meals. Issued by the State Health and Family Planning Commission of the people’s republic of China WS/T 554-2017), daily nutrient and food intake were evaluated.

### Blood sample collection and detecting

Blood sample were collected to detect the concentration of retinol by high performance liquid chromatography method, and we judged marginal deficiency and deficiency of vitamin A among children and adolescents by boundary values of serum retinol level (15). The serum retinol content of children and adolescents is less than 0.2 ng/ml, it is determined as vitamin A deficiency. Blood sample were collected to detect the concentration of 25-hydroxyvitamin D by enzyme linked immunosorbent assay, and according to Standards recommended by the American Endocrine Association, we judged deficiency and inadequacy of vitamin D among children and adolescents by boundary values of the concentration of 25-hydroxyvitamin D (16). If the content of serum 25 hydroxyvitamin D in children and adolescents is less than 20 ng/ml, it is determined as vitamin D deficiency.

### Statistical analysis

As continuous variables were not normally distributed, they were described as the median, 25th and 75th percentiles. The differences of daily food intake and daily dietary nutrient intake between children and adolescents living in urban area and rural area were evaluated by the non-parametric test (Kruskal-Wallis test). Data processing and statistical analyses were performed using SAS 9.2 software (SAS Institute, Cary, NC, USA). All tests were two-sided, and the level of significance was set at *p* < 0.05.

## Results

### Study population

A total of 1,421 children and adolescents aged 6–17 years were included in the study, including 734 (51.6%) in rural area (378 males and 356 females) and 687 (48.3%) in urban area (336 males and 351 females). The number of children and adolescents aged 6–8, 9–11, 12–14, and 15–17 years were 338 (23.8%), 284 (20.0%), 363 (25.5%), 436 (30.7%), respectively. The prevalence of obesity was 10.5% among children and adolescents living in urban area, higher than those living in rural area (5.9%) (*Z* = 10.192, *P* = 0.001). The prevalence of overweight was 26.1% among children and adolescents living in urban area, higher than those living in rural area (15.8%) (*Z* = 22.671, *P* = 0.000).

### Disparity in serum vitamin A, D and serum zinc concentration in urban and rural children and adolescents

Serum vitamin A level of children and adolescents in rural area was lower than that of children and adolescents in urban area (*Z* = 7.082, *P* < 0.05), while the vitamin D level was higher than that of children and adolescents in urban area (*Z* = −4.967, *P* < 0.05). The serum zinc level of rural children and adolescents was higher than that of urban adolescents (*Z* = 3.906, *P* < 0.05) (Table 1). Serum vitamin A level of children and adolescents aged 9–14 years in rural area was lower than that of children and adolescents in urban area (*P* = 0.000). Serum vitamin D level of children and adolescents aged 9–17 years in rural area was higher than that of children and adolescents in urban area (*P* < 0.05). The serum zinc level of adolescents aged 12–17 years in rural area was higher than that of urban adolescents (*P* < 0.05).

**TABLE 1:** Serum vitamin A, D and serum zinc median concentration of urban and rural children and adolescents.

Age (years)		Urban area [median (p25, p75)]	Rural area [median (p25, p75)]	*Z*	*p*
Total	Vitamin A (ng/ml)	0.38 (0.32, 0.45)	0.34 (0.27, 0.41)	7.082	0.000
	Vitamin D (mg/L)	21.25 (16.64, 26.84)	23.02 (18.46, 28.97)	-4.967	0.000
	Zinc (ug/dl)	101.00 (89.60, 120.00)	107.00 (92.80, 126.00)	-3.906	0.000
6–8	Vitamin A (ng/ml)	0.32 (0.25, 0.38)	0.30 (0.25, 0.37)	1.320	0.187
	Vitamin D (mg/L)	24.71 (20.42, 29.03)	24.25 (20.48, 31.21)	-0.451	0.652
	Zinc (ug/dl)	111.00 (97.40, 124.75)	112.00 (96.65, 125.75)	-0.710	0.478
9–11	Vitamin A (ng/ml)	0.37 (0.30, 0.43)	0.32 (0.27, 0.38)	3.859	0.000
	Vitamin D (mg/L)	22.13 (18.76, 27.37)	24.91 (19.35, 30.31)	-1.985	0.047
	Zinc (ug/dl)	105.00 (88.95, 125.00)	106.00 (91.20, 123.00)	-0.215	0.830
12–14	Vitamin A (ng/ml)	0.41 (0.35, 0.48)	0.34 (0.27, 0.41)	6.445	0.000
	Vitamin D (mg/L)	20.33 (16.32, 26.21)	22.62 (17.30, 27.98)	-2.191	0.028
	Zinc (ug/dl)	99.60 (89.70, 115.00)	109.00 (93.40, 123.50)	-2.787	0.005
15–17	Vitamin A (ng/ml)	0.39 (0.34, 0.47)	0.38 (0.30, 0.46)	1.190	0.234
	Vitamin D (mg/L)	19.00 (13.41, 24.44)	21.43 (18.06, 26.83)	-4.913	0.000
	Zinc (ug/dl)	97.20 (84.35, 117.00)	106.00 (89.35, 132.50)	-3.411	0.001

### Nutrient intake of children and adolescents in rural area and comparison with the recommended values

**FIGURE 1:**
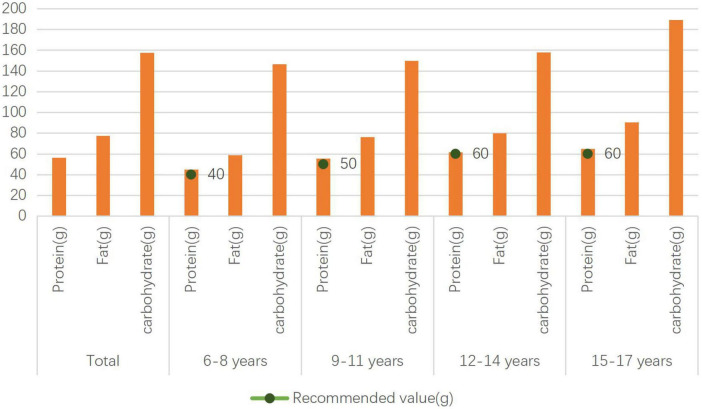
Protein, fat, and carbohydrate intake of rural children and adolescents.

The protein intake of rural children aged 6–8, 9–11, 12–14, 15–17 years, was higher than the recommended value of 40, 50, 60, and 60 g/d, respectively (Figure 1).

The dietary fiber intake of rural children and adolescents aged 6–8, 9–11, 12–14, 15–17 years, was lower than the recommended value of 20, 20, 20, and 25 g/d, respectively, and the calcium intake of that was lower than the recommended value of 750, 850, 950, and 800 g/d.

Vitamin B1, vitamin C, vitamin A, vitamin B2 intake of rural children aged 6–8 years was lower than the recommended value of 0.9 mg/d, 60 mg/d, 450 μg RE/d, and 0.9 mg/d, respectively. Vitamin B1, vitamin C, vitamin A, vitamin B2 intake of rural children aged 9–11 years was lower than the recommended value of 1.1 mg/d, 75 mg/d, 550 μg RE/d, and 1.1 mg/d, respectively. Vitamin B1, vitamin C, vitamin A, vitamin B2 intake of rural adolescents aged 12–14 years was lower than the recommended value of 1.2 mg/d, 95 mg/d, 630 μg RE/d, and 1.2 mg/d, respectively. Vitamin B1, vitamin C, vitamin A, vitamin B2 intake of rural adolescents aged 12–14 years was lower than the recommended value of 1.3 mg/d, 100 mg/d, 630 μg RE/d, and 1.3 mg/d, respectively (Table 2).

**TABLE 2:** Dietary fiber, calcium, vitamin B1, vitamin C, vitamin A, vitamin B2 intake of urban and rural children and adolescents.

Daily intake	Urban area			Rural area			*Z*	*p*
	Median	25%	75%	Median	25%	75%		
Dietary fiber (g)	6.16	3.71	9.94	6.63	4.24	10.63	-2.339	0.019
Calcium (mg)	367.87	240.78	513.44	368.60	247.27	524.75	-0.489	0.625
Vitamin C (mg)	35.82	22.02	56.84	43.24	27.03	69.38	-4.781	0.000
Vitamin B1 (mg)	0.68	0.50	0.93	0.67	0.50	0.90	0.612	0.540
Vitamin B2 (mg)	0.73	0.55	1.02	0.70	0.52	1.00	1.442	0.149
Vitamin A (ug RE)	264.56	171.01	419.34	236.25	145.07	413.14	2.344	0.019

### Various food intake of children and adolescents in rural area and comparison with the recommended value

The livestock, poultry, and meat intake of rural children and adolescents aged 6–8, 9–11, 12–14, 15–17 years, was higher than the recommended value of 40, 50, 60, and 75 g/d, respectively, while the vegetable intake of rural children and adolescents aged 6–8, 9–11, 12–14, 15–17 years, was lower than the recommended value of 300, 350, 400, and 450 g/d, respectively.

**FIGURE 2:**
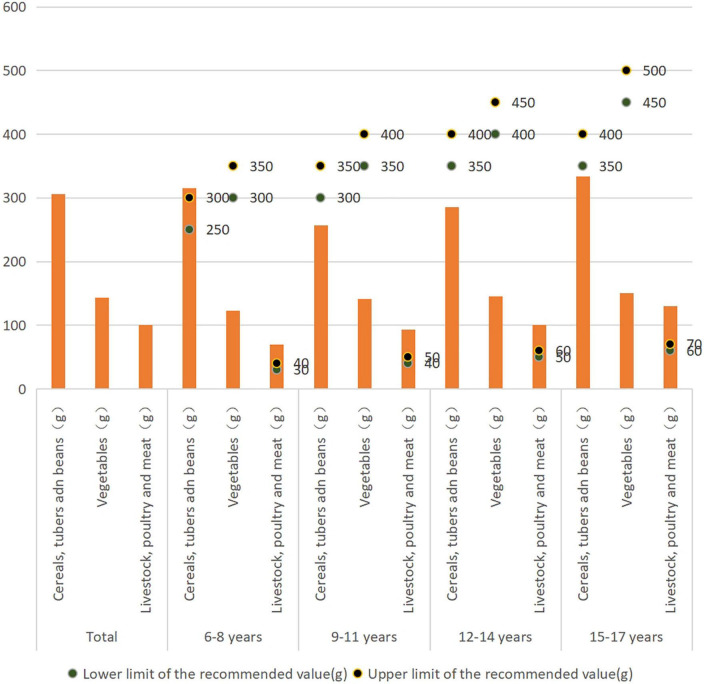
Cereals, tubers and beans, vegetables and livestock, poultry and meat intake of rural children and adolescents.

Fruits, eggs, Dairy, and Soybean and nuts intake of rural children aged 6–8 years was lower than the recommended value of 150, 50, 200, and 30 g/d, respectively. Cereals, tubers and beans, fruits, eggs, Dairy, and Soybean and nuts intake of rural children aged 9–11 years was lower than the recommended value of 300, 200, 200, 200, and 35 g/d, respectively. Fruits, eggs, Dairy, and Soybean and nuts intake of rural children aged 12–14 years was lower than the recommended value of 250, 75, 250, and 40 g/d, respectively. Fruits, eggs, Dairy, and Soybean and nuts intake of rural children aged 15–17 years was lower than the recommended value of 300, 75, 250, and 50 g/d, respectively (Figure 2).

### Edible oil and salt intake of rural children and adolescents and comparison with the recommended values

**FIGURE 3:**
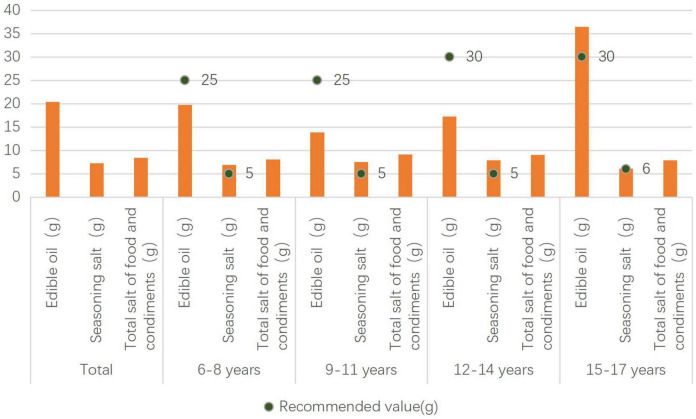
Edible oil and seasoning salt intake of rural children and adolescents.

The seasoning salt intake of rural children aged 6–14 years was higher than the recommended value of 5 g/d. The edible oil intake of rural adolescents aged 15–17 years is higher than the recommended value of 30 g/d, and the seasoning salt intake (Figure 3).

### Medium and high intensity exercise time and sedentary time of rural children and adolescents

Rural adolescents aged 12–17 years spend more time on moderate and high intensity sports than urban adolescents. The sedentary time of rural adolescents aged 12–17 years is less than that of urban children and adolescents (*p* < 0.05) (Table 3). There was correlation between medium and high exercise time and vitamin D levels (Spearman’s rho = 0.064, *p* = 0.019).

**TABLE 3:** Medium and high intensity exercise time and sedentary time of urban and rural adolescents.

Age (years)		Urban area	Rural area	*Z*	*p*
Total	Medium and high intensity exercise time (h)	0.5	0.54	4.832	0.000
	Sedentary time (h)	8.93	7.65	-7.482	0.000
6–8	Medium and high intensity exercise time (h)	0.50	0.58	-1.397	0.163
	Sedentary time (h)	6.71	6.57	1.142	0.254
9–11	Medium and high intensity exercise time (h)	0.50	0.50	-0.083	0.934
	Sedentary time (h)	7.36	6.60	2.767	0.006
12–14	Medium and high intensity exercise time (h)	0.50	0.83	-4.323	0.000
	Sedentary time (h)	9.71	8.00	4.989	0.000
15–17	Medium and high intensity exercise time (h)	0.50	0.50	-3.595	0.000
	Sedentary time (h)	11.29	9.64	5.694	0.000

## Discussion

Nutrition is the material basis for ensuring the growth and development of children and adolescents and maintaining their health. Childhood and adolescence is the key period to acquire nutrition knowledge, establish healthy habits and develop a healthy lifestyle for life. The purpose of this study is to analyze the nutrition status of rural children and adolescents, so as to understand the nutrition and health problems of them. We want to provide evidence for rural children and adolescents to obtain scientific nutrition and health knowledge, and to develop good dietary behavior.

Vitamin A is a micro essential nutrient, mainly used for vision system, growth and development, maintenance of epithelial cell integrity, immune function and reproduction (17). Insufficient intake of vitamin A is the main source of vitamin A deficiency. Vitamin A deficiency reduces immunity and leads to increased morbidity and mortality from night blindness, corneal ulcer, keratitis, dry eye, and related ocular symptoms (18). Vitamin A deficiency increases mortality from measles, diarrhea, and respiratory diseases (19). Attention should be paid to the problem of vitamin A deficiency in children and adolescents. This study found that the serum vitamin A level of rural children and adolescents was lower than that of urban children and adolescents. It is suggested to increase the types of food and the intake of foods rich in vitamin A for rural children and adolescents. Vitamin A in food comes from liver and cod liver oil, dairy products, egg yolk. Vitamin A carotenoids in plant foods, such as dark green leafy vegetables, dark yellow and orange vegetables and fruits, are good food sources (20), however, the bioavailability and biotransformation rate of vegetables are low (21).

Vitamin D deficiency is associated with non-communicable and infectious diseases (22). New lifestyle habits, the current global “epidemic” of obesity among children and adolescents, and other preventable risk factors may play a role in promoting the occurrence of vitamin D deficiency (23). The determination of serum 25 hydroxyvitamin D (25 [OH] D) is widely accepted as a substitute indicator of vitamin D status (24). Vitamin D is obtained mainly by exposure to sunlight, and many determinants of vitamin D status are related to sunlight exposure (25, 26). We found that there was correlation between medium and high exercise time and vitamin D levels and the level of vitamin D in rural area was higher than that in urban children and adolescents, which may be related to the more opportunities for rural children and adolescents to receive sunlight exposure.

Zinc is an essential trace element for children’s growth and development, but zinc deficiency is a serious nutritional problem all over the world. In the past 10 years, in addition to zinc intake, the zinc status of rural school-age children in China has also been significantly improved. However, in 2012, poor rural area in China still suffered from zinc deficiency (27). This study found that the serum zinc level of rural children and adolescents is higher than that of urban children and adolescents, and the specific reasons for the difference need to be further explored. Zinc in human body is mainly obtained through diet. To prevent zinc deficiency, we should stick to a balanced diet, eat some red meat with rich zinc content, some seafood (such as oysters, but not large quantities).

### Nutrient, food, edible oil, and salt intake of rural children and adolescents

Nutrition transformation describes changes in diet and nutrition driven by economic, social and demographic changes (28). In the early transition phase, these changes can improve food security and health, but are also associated with an increase in chronic non-infectious diseases (29). Thomas found that staying away from high fiber, low fat, low salt and low processed sugar diet is a significant health risk for people in transition (28). Rosinger reported that the increase in household expenditure on food on the market was related to a moderate increase in BMI, body fat and the probability of overweight or obesity (29). In this study, we found that the protein intake of rural children and adolescents met the recommended values, but there was still a gap between the intake of dietary fiber, calcium, vitamin B1, vitamin C, vitamin A, and vitamin B2 and the recommended values. In terms of food types, the intake of vegetables, fruits, eggs, milk, soybean nuts, fish and shrimp was lower than the recommended value, and the intake of dark vegetables in rural area was lower than that in urban area. The meat of livestock and poultry is higher than the recommended value. The seasoning salt intake for rural children aged 6–14 years was higher than the recommended value of 5 g/d. The edible oil intake of rural adolescents aged 15–17 years was higher than the recommended value of 30 g/d, and much higher than that of urban adolescents. We should strengthen the publicity of dietary nutrition knowledge for rural children and adolescents, and let rural children and adolescents actively learn food nutrition and learn healthy living style.

Children and adolescents should learn to choose food, and read food labels when choosing prepackaged food. They should choose foods with high nutrient density through food labels, and try to avoid foods with high edible oil, salt and sugar. For overweight and obese children and adolescents, it is necessary to adjust the dietary structure to control the total energy intake.

### Physical activity and sedentary time of rural children and adolescents

Physical activity has many physical and psychosocial benefits. However, lifestyle changes, including reduced opportunities for physical activity in a variety of environments, have led to the escalation of overweight and obesity and related health problems (29). Lack of exercise and malnutrition are strong predictors of negative health conditions and increase the risk of obesity and other chronic diseases (30). Regular physical activity, sufficient sleep and reduced sedentary time can strengthen bones and muscles, and improve cardiopulmonary function to promote growth and development and prevent overweight and obesity, as well as improve learning efficiency. This study found that rural adolescents aged 12–17 years spent more time in high-intensity exercise than urban adolescents, and except for the 6–8 age group. The rural children and adolescents have less time to sit still than the urban adolescents. The physical activity of rural children and adolescents was slightly better than that of urban adolescents, but it is still insufficient. We suggest that children and adolescents should have accumulate at least 60 min of high-intensity physical activity every day, and at least three times a week. We give priority to outdoor activities if conditions permit.

### Strengths and limitations

The main strength of our study was that we collected the dietary data by home and school visits spanning 3 days through interviews with each child of the sampling schools, including breakfast, lunch, dinner and the intake of extra meals or snacks. All the foods and condiments and edible oil that intake during the three consecutive days of 24-h were recorded. Another strengths was that we calculated the daily person energy and nutrient intake with the China Food Composition Table, thus we have the detailed food and nutrient intake data. Our study also had several limitations. Firstly, Family information of the children and adolescents were not available in this study. Secondly, blood samples were collected in 2016–2017, and seasonal factors are not considered. We didn’t take seasonal factors into account when comparing the serum 25(OH)D concentrations between rural and urban areas. The analysis of disparity of demographics and seasonal factors on nutritional status should be further explored in the future study.

## Conclusion

The results show that the overweight rate and obesity rate of rural children and adolescents are 15.8 and 5.9% respectively, and obesity has begun to spread to the countryside. Among the food categories, the intake of vegetables, fruits, eggs, milk, soybean nuts, fish, and shrimp was lower than the recommended values, but the intake of livestock and poultry meat was higher than the recommended value. The intake of condiment salt and edible oil is high. Comprehensive measures should be taken to promote the nutritional health of rural children and adolescents. We should strengthen the publicity of dietary nutrition knowledge for rural children and adolescents, so that they can understand food and the role of food in maintaining their health and preventing diseases.

## Data availability statement

The raw data supporting the conclusions of this article will be made available by the authors, without undue reservation.

## Ethics statement

The study was approved by the Ethical Committee of Zhejiang Provincial Center for Disease Control and Prevention (201614). All information acquired was kept confidential and was only accessible by the researchers.

## Author contributions

YZ and R-HZ were responsible for the study design. YZ was responsible for data collection and analysis and manuscript writing and revision. L-CH, DS, DZ, and M-JH took part in the field investigation and data collection. All authors contributed to the article and approved the submitted version.
